# Oral Health Literacy and Oral Health-Related Quality of Life Among Peruvian Children

**DOI:** 10.21142/2523-2754-1404-2026-310

**Published:** 2026-10-01

**Authors:** César Casapaico-Cucche, Teresa Evaristo-Chiyong, Silvia Luza-Montero

**Affiliations:** 1 Dental surgeon. Faculty of Dentistry, Universidad Nacional Mayor de San Marcos. Lima, Peru. cesarcasapaicoc@gmail.com Universidad Nacional Mayor de San Marcos Dental surgeon Faculty of Dentistry Universidad Nacional Mayor de San Marcos Lima Peru cesarcasapaicoc@gmail.com; 2 PhD. SAETA Research group. Faculty of Dentistry, Universidad Nacional Mayor de San Marcos. Lima, Peru. tevaristoc@unmsm.edu.pe Universidad Nacional Mayor de San Marcos SAETA Research group Faculty of Dentistry Universidad Nacional Mayor de San Marcos Lima Peru; 3 PhD. ACOB Research group. Faculty of Dentistry, Universidad Nacional Mayor de San Marcos. Lima, Peru. sluzam@unmsm.edu.pe Universidad Nacional Mayor de San Marcos ACOB Research group Faculty of Dentistry Universidad Nacional Mayor de San Marcos Lima Peru sluzam@unmsm.edu.pe

**Keywords:** health literacy, health-related quality of life, oral health, parent-child relationship, non-governmental organization, alfabetización en salud, calidad de vida relacionada con la salud, salud bucal, interacción padres-hijos, organización no gubernamental

## Abstract

**Objective:**

To determine the relationship between the level of oral health literacy among parental caregiver and the oral health-related quality of life of 11- and 12-year-old children belonging to a non-governmental organization in Peru.

**Materials and methods:**

An observational, cross-sectional, and correlational study was conducted on a sample of 208 caregiver-child pairs. Oral health literacy was assessed using the HeLD-14 instrument, and oral health-related quality of life (OHRQoL) using the CHILD-OIDP questionnaire, both validated in the Peruvian population. Inferential analysis was performed using the nonparametric Mann-Whitney U test, Kruskal-Wallis test, and Spearman’s correlation. A Poisson regression model with robust variances was applied, for which the variable “impact on quality of life” was dichotomized. A significance level of 5% was considered.

**Results:**

The average oral health literacy score of the caregiver parents was 27.2 ± 7.6. A predominantly severe impact (48.56%) was observed, followed by a moderate impact (38.46%) on children’s oral health-related quality of life. A strong negative correlation (Rho = -0.793; p < 0.05) was found between the oral health literacy of the parent caregiver and the child’s HRQoL. A PR of 0.88 was obtained; 95% CI [0.86-0.89]; p < 0.001.

**Conclusion:**

As the primary caregiver’s oral health literacy increases, the severity of the impact on children’s oral health-related quality of life decreases.

## Resumen

**Objetivo:**

Determinar la relación entre el nivel de alfabetización en salud oral del cuidador parental y la calidad de vida relacionada con la salud bucal de niños de 11 y 12 años pertenecientes a una ONG del Perú.

**Materiales y métodos:**

Estudio observacional, transversal y analítico correlacional en una muestra de 208 binomios cuidador-niño. El nivel de alfabetización en salud oral se evaluó mediante el instrumento HeLD-14, y la calidad de vida relacionada con la salud bucal (CVRSB) con el cuestionario CHILD-OIDP validados en población peruana. El análisis inferencial se realizó con las pruebas no paramétricas U de Mann-Whitney, Kruskal-Wallis y correlación de Spearman. Se aplicó el modelo de regresión de Poisson con varianzas robustas, para lo cual la variable impacto en la calidad de vida fue dicotomizada. Se consideró un nivel de significancia del 5%.

**Resultados:**

El puntaje promedio de alfabetización en salud oral de los padres cuidadores fue 27.2 ± 7.6. Se evidenció un impacto principalmente severo (48.56%), seguido por uno regular (38.46%), en la calidad de vida relacionada con la salud bucal de los niños. Se halló una correlación negativa fuerte (Rho = −0.793; p < 0.05) entre la alfabetización en salud oral del padre cuidador y la CVRSB del niño. Se obtuvo un RP = 0.88; IC95%: 0.86-0.89; p < 0.001.

**Conclusión:**

Al aumentar el nivel de alfabetización en salud oral del padre cuidador, disminuye la severidad del impacto en la calidad de vida relacionada a la salud bucal de los niños.

## INTRODUCTION

Child oral health remains a significant global public health concern.^1^^)^ According to the World Health Organization, health is a state of complete physical, mental, social well-being, and not merely the absence of disease, which underscores the need to address oral health from a comprehensive perspective.^2^

Oral health-related quality of life (OHRQoL) is recognized as an indicator that allows the assessment of the functional, emotional, and social impact of oral conditions from the individual’s perspective.^3^ As such, it complements conventional clinical measures used in dental practice and research, providing a more comprehensive framework for assessing oral health at both individual and population levels.^4^

Various social determinants influence child oral health, including the family environment and the competencies of the primary caregiver.^5^^-^^6^ Health literacy has been identified as a key factor in adopting preventive behaviors and health-related decision-making. In the dental field, oral health literacy (OHL) is defined as the ability to obtain, understand, and use oral health-related information to make appropriate decisions. ^7^ Previous studies have reported that low levels of parental literacy are associated with greater caries experience, lower adherence to preventive practices, and limited use of dental services.^8^^-^^10^ A scoping review indicated that when parental literacy is low, children tend to have a higher risk of caries and poorer OHRQoL outcomes. Parental influence is particularly relevant during middle childhood, defined as the period between ages 6 and 12, when children begin to develop autonomy but still depend on the supervision and guidance of their caregivers to maintain healthy habits. During this stage, the consolidation of oral hygiene practices and dietary patterns may determine the future risk of oral disease.^11^

Divaris ^5^ and Zhang ^12^ found that parental oral health literacy influences preschool-aged children’s quality of life. In contrast, other studies^13^^-^^14^ have reported no significant association between these variables. Although some studies have explored the association between parental OHL and children’s OHRQoL, the available evidence remains limited and heterogeneous, particularly with respect to specific age groups such as middle childhood.

Furthermore, the majority of studies have predominantly focused on objective clinical indicators, with comparatively less emphasis on children’s oral health-related quality of life and its potential association with parental oral health literacy. This gap is particularly evident in Latin American settings and among socially vulnerable populations, where inequalities in access to information and health services can exacerbate the impact of social determinants.^15^ Therefore, further research is needed to clarify this relationship in these contexts. Addressing the association between parental OHL and children’s OHRQoL during middle childhood is of particular importance from both clinical and public health perspectives.

Understanding this relationship will help identify potential gaps in health education, guide preventive interventions targeting caregivers, and contribute to the design of strategies that promote not only improved clinical indicators but also greater overall child well-being.

Thus, the present study aimed to determine the relationship between the level of parental caregiver OHL and OHRQoL in 11- and 12-year-old children belonging to a non-governmental organization that serves families in vulnerable situations in Lima and Callao, Peru. The hypothesis was that lower levels of caregiver OHL are associated with poorer OHRQoL in children.

## MATERIALS AND METHODS

An observational, cross-sectional, and correlational study was conducted. The study was approved by the Ethics Committee of the Faculty of Dentistry at Universidad Nacional Mayor de San Marcos, with registration number 019-CEI-FO-2025. 

The study population consisted of 228 child-parent caregiver pairs of 11- and 12-year-old children affiliated with the non-governmental organization “Corazones Felices” in Peru, which serves populations in Lima and Callao. Census sampling was conducted, and participants were selected per the established inclusion and exclusion criteria. The unit of analysis was the child-parent caregiver pair.

Parents responsible for the care of 11- and 12-year-old children belonging to the organization were included in the study. Pairs were excluded if the parent or the child was not in an adequate physical or mental condition to complete the questionnaires.

Written informed consent was obtained from the caregiver parents, and informed assent was obtained from the children prior to instrument administration, ensuring confidentiality and anonymity of the information.

The level of parental caregiver OHL was assessed using the Health Literacy in Dentistry-14 (HeLD-14) instrument, which was previously adapted and validated in a Peruvian population.^16^ This instrument consists of 14 items distributed across seven dimensions (receptivity, understanding, support, economic barriers, access, communication, and utilization), with a Likert-type scale ranging from 0 to 4 points. The total score ranges from 0 to 56, with lower scores indicating lower levels of OHL.

Children’s OHRQoL was measured using the Child Oral Impacts on Daily Performances (Child-OIDP) questionnaire, Spanish version validated in Peru.^17^ The instrument evaluates self-perceived oral conditions over the past three months and their impact on eight daily activities. For each activity or performance, the severity and frequency of impact were multiplied to obtain an intensity score per activity. The sum of the scores across the eight activities allowed for the estimation of the overall impact intensity, expressed as a percentage of the maximum possible score (72 points). The level of impact was classified into five categories: 0.1-11.1% (very low intensity), 11.2-22.2% (low intensity), 23.3-44.4% (moderate intensity), 44.5-66.7% (severe intensity), and 66.8-100% (very severe intensity). Additionally, a sociodemographic questionnaire was administered to the caregiver parent, including sex, age, education level, occupation, and income level.

A pilot study was conducted with 24 child-caregiver pairs to assess the reliability of the instruments used. The HeLD-14 questionnaire demonstrated adequate internal consistency, with Cronbach’s alpha coefficient of 0.87. In the case of the Child-OIDP questionnaire, reliability was assessed using the test-retest method, administering it to the same children one week later. The degree of agreement between the scores obtained for each of the eight daily activities was determined using the intraclass correlation coefficient (ICC), yielding values ranging from 0.81 to 0.99.

Data collection was carried out through visits to community clubs and households registered in the database provided by the NGO. The questionnaires were administered by the principal investigator. Before analysis, the questionnaires were reviewed, and incomplete ones were excluded.

Descriptive analysis was performed using frequency tables for categorical variables and measures of central tendency and dispersion for quantitative variables. Due to the ordinal nature of the variables and data distribution, inferential analysis was conducted through nonparametric tests: the Mann-Whitney U test for comparisons between two independent groups, the Kruskal-Wallis test for comparisons among more than two groups, and Spearman’s rank correlation coefficient to assess the relationship between OHL and OHRQoL. A Poisson regression model with robust variance estimation was employed; for this purpose, the quality-of-life impact variable was dichotomized, and all caregiver sociodemographic variables were included in the adjusted model. A statistical significance level of 5% (p < 0.05) was considered. 

## RESULTS

A total of 208 children and their respective parental caregivers were evaluated in the study, yielding a response rate of 91.2%. The most frequent sociodemographic characteristics were female sex (77.40%; n = 161), completion of secondary education (81.25%; n = 169), self-employment (41.83%; n = 87), and income below the average monthly minimum wage (66.83%; n = 139) (Table 1).

**Table 1:** Sociodemographic characteristics of parental caregivers of 11- and 12-year-old children

Sociodemographic characteristics		n	%
Total, sample		208	100
Sex	Male	47	22.6
	Female	161	77.4
Age (years)	18-30	2	0.96
	31-40	93	44.71
	41-50	94	45.19
	≥51	19	9.13
Education level	Primary	18	8.65
	Secondary	169	81.25
	Higher education	21	10.10
Occupation	Dependent worker	37	17.79
	Self-employment	87	41.83
	Homemaker-not working	84	40.38
Income level	Below MMW	139	66.83
	MMW	35	16.83
	> MMW	34	16.35

MMW = Monthly minimum wage

Parental caregiver OHL had a mean score of 27.2 ± 7.6. According to sociodemographic characteristics, OHL scores were highest among males (31.51 ± 8.95; p < 0.001) and in the 31-40 age group (28.76 ± 8.38), while the lowest scores were observed among those older than 51 years (23.79 ± 7.99; p = 0.039). Regarding educational level, individuals with higher education had the highest mean score (34.38 ± 8.34; p < 0.001). In terms of occupation, dependent workers exhibited the highest scores (30.78 ± 7.49), whereas homemakers or those not working had the lowest (24.86 ± 6.99; p < 0.001). Caregiver parents with income above the minimum wage had the highest mean score (38.79 ± 3.96; p < 0.001) (Table 2).

**Table 2:** Oral health literacy score (HeLD-14) according to sociodemographic variables of parental caregivers

Sociodemographic characteristics		HeLD-14			Statistical test
		Mean	SD	Median	
Sex	Male	31.51	8.95	31.00	Mann- Whitney U p <0.001
	Female	25.97	6.70	25.00	
Age(years)	18 a 30	25.50ab	13.44	25.50	Kruskal-Wallis p = 0.039
	31 a 40	28.76a	8.38	27.00	
	41 a 50	26.43ab	6.30	25.50	
	> 51	23.79b	7.99	20.00	
Education level	Primary	19.28a	3.58	19.00	Kruskal-Wallis p < 0.001
	Secondary	27.18b	7.16	26.00	
	Higher education	34.38c	6.84	36.00	
Occupation	Dependent worker	30.78a	7.49	30.00	Kruskal-Wallis p < 0.001
	Self-employment	27.99ab	7.56	27.00	
	Homemaker-not working	24.86b	6.99	24.00	
Income level	Below MMW	25.23a	6.49	24.00	Kruskal-Wallis p < 0.001
	MMW	25.83a	5.65	26.00	
	> MMW	36.79b	6.39	36.00

SD = Standard deviation. Different letters indicate statistically significant differences after Dunn’s post hoc test with Bonferroni correction. MMW= Monthly minimum wage

The oral conditions most frequently perceived as problematic by children, with a prevalence over 60%, were bleeding gums (91.35%), toothache (86.06%), bad breath (85.10%), tooth sensitivity (74.52%), dental calculus or tartar (64.90%), malpositioned teeth (62.02%), oral sores or ulcers (61.06%), and altered tooth shape or size (60.10%) (Table 3).

**Table 3:** Frequency of self-perceived oral conditions reported as problems by 11- and 12-year-old children

Oral condition	No.		Yes	
	n	%	n	%
Toothache	29	13.94	179	86.06
Tooth sensitivity (e.g., cold, heat, or sweets)	53	25.48	155	74.52
Decayed tooth (cavitated or damaged)	93	44.71	115	55.29
Loss of a primary tooth	116	55.77	92	44.23
Spacing between teeth	117	56.25	91	43.75
Permanent tooth fracture	127	61.06	81	38.94
Tooth color	92	44.23	116	55.77
Tooth shape or size	83	39.90	125	60.10
Malpositioned teeth (e.g., crooked, protruding, crowded)	79	37.98	129	62.02
Bleeding gums	18	8.65	190	91.35
Gingival inflammation	111	53.37	97	46.63
Dental calculus	73	35.10	135	64.90
Oral sores or ulcers	81	38.94	127	61.06
Bad breath	31	14.90	177	85.10
Facial or oral deformity	185	88.94	23	11.06
Eruption of permanent teeth	130	62.50	78	37.50
Loss of permanent teeth	159	76.44	49	23.56

The impact of oral conditions on quality of life was mainly perceived as severe (n = 101; 48.56%) and moderate (n = 80; 38.46%). No differences were found in the perception of impact according to sex (p = 0.504) or age of the child (p = 0.602) (Table 4).

**Table 4:** Impact of oral conditions on children's quality of life according to sex and age

Characteristic		Low impact		Moderate impact		Severe impact		Very severe impact		Total	p-value
		n		%	n	%	n	%	n	%	n	%	
Sex	Male	5	2.40	39	18.75	43	20.67	4	1.92	91	43.75	0.504
	Female	12	5.77	41	19.71	58	27.88	6	2.88	117	56.25	
Age (years)	11	6	2.88	38	18.27	52	25.00	5	2.40	101	48.56	0.662
	12	11	5.29	42	20.19	49	23.56	5	2.40	107	51.44	
Total, sample		17	8.17	80	38.46	101	48.56	10	4.81	208	100.00	

Test: Chi-square

A strong, negative, and statistically significant correlation was observed between parental caregiver OHL (HeLD-14) scores and the impact of oral conditions on children’s quality of life (rho = -0.793; p < 0.001), which indicates that higher levels of parental OHL are associated with lower severity of the impact of oral conditions on children’s quality of life.

The quality-of-life impact variable was dichotomized (low-moderate impact vs. severe-very severe impact). The model was adjusted considering all caregiver sociodemographic characteristics. Multicollinearity among the variables included in the model was assessed, yielding an average variance inflation factor of 5.14. In the multivariate analysis, adjusted for all caregiver sociodemographic variables included in the model, only OHL scores showed a statistically significant association with quality-of-life impact. Specifically, for each one-point increase in literacy score, the prevalence of negative impact on quality of life decreased by 12% (PR = 0.88; 95% CI: 0.86-0.89; p < 0.001) (Table 5).

**Table 5:** Adjusted multivariate analysis between sociodemographic characteristics, oral health literacy, and impact on quality of life

Caregiver characteristics	Impact on quality of life			
	PR	CI 95%		p
Sex				
Female (ref. Male)	1.32	0.98	1.79	0.071
Age (years)				
41 a 50 (ref. 18-40)	1.09	0.87	1.36	0.459
≥51 (ref. 18-40)	0.92	0.66	1.29	0.625
Education level				
Secondary (ref. Primary)	1.06	0.80	1.39	0.699
Higher education (ref. Primary)	1.17	0.66	2.08	0.585
Income				
≥ MMW (ref. < MMW)	0.98	0.74	1.30	0.899
Occupation				
Self-employment (ref. dependent)	0.97	0.67	1.42	0.892
Homemaker / not working (ref. dependent)	0.69	0.47	1.00	0.050
Oral health literacy score	0.88	0.86	0.89	<0.001

PR: Prevalence ratio. Model: Poisson regression with robust variance. MMW = Monthly minimum wage

## DISCUSSION

OHL has become a key component in public health, directly influencing understanding, decision-making, and the adoption of preventive behaviors related to oral well-being.^18^

The results of the present study showed a mean OHL score of 27.2 ± 7.6 out of a maximum possible score of 56, with predominant difficulties in domains related to economic barriers and access to dental care. Additionally, a strong and statistically significant negative correlation (rho = −0.793; p < 0.001) was identified between parental caregiver OHL and the impact of oral conditions on children’s quality of life. This indicates that higher caregiver literacy is associated with a lower perceived negative impact on children.

These findings are consistent with prior evidence that caregivers’ OHL plays a determinant role in children’s oral health outcomes. Baskaradoss ^10^ observed that children of parents with low literacy levels had greater dental caries experience, while Ristow et al. ^19^ and Yazdani et al. ^20^ also found a direct relationship between low parental literacy and a higher prevalence of untreated caries or higher DMFT indices in children. Similarly, Bridges et al. ^8^^)^ emphasized that caregivers’ understanding of dental information influences the adoption of protective habits in children. The findings of this study reinforce this pattern in a school-aged population within a socially vulnerable context.

Regarding sociodemographic characteristics, male caregivers exhibited higher levels of OHL. This finding differs from those reported by Mahmud ^21^ and Orozco ^22^, who found higher literacy levels among women. Evidence suggests that other social characteristics (educational level, economic factors, among others) may influence literacy, and interpreting the effect of caregiver sex alone may be insufficient without considering these additional variables.^23^

In terms of educational level, caregivers with higher education achieved significantly higher literacy scores. This is consistent with findings reported by Baskaradoss ^10^ and Yazdani ^20^, who argue that OHL is closely linked to educational attainment, as it facilitates the understanding of dental concepts and the application of preventive recommendations. The positive association between education level and OHL suggests that the comprehension of dental information largely depends on cognitive skills acquired through formal education, enabling individuals to interpret and apply professional recommendations more effectively.^24^

With respect to occupation, caregivers with dependent employment demonstrated higher literacy levels compared to those engaged in household duties or unemployed. Baskaradoss ^10^ indicate that more stable and formal employment often provides greater access to knowledge and health services, which positively influences literacy. Furthermore, studies such as Maito et al. ^25^ show that lower educational attainment and informal employment limit the ability to make informed decisions regarding oral health, which is consistent with the findings of the present study.

Regarding income level, caregivers with greater economic resources achieved higher literacy scores. This aligns with findings reported by Mahmud ^21^, Orozco ^22^, and Baskaradoss ^26^, who demonstrated the impact of economic factors on literacy, considering that access to dental services, educational materials, and preventive practices largely depends on the family’s financial capacity. In this study, caregivers with lower income reported difficulties in affording dental consultations or medications, reinforcing trends observed in previous research.

The analysis of self-perceived oral conditions among children revealed a high prevalence of problems such as gingival bleeding, dental pain, halitosis, and tooth sensitivity, which negatively affected their daily activities, particularly eating and performing oral hygiene. These findings are consistent with those reported by Clavijo and Campos,^24^ who found that children of parents with lower oral health knowledge exhibited poorer hygiene habits. Additionally, these results suggest that parental literacy influences not only access to dental care but also the development of preventive behaviors within the household.

Regarding the severity of the impact on OHRQoL, most children experienced a moderate to severe level of impact, which highlights that oral health problems significantly affect their well-being. Similarly, findings from a systematic review have demonstrated the negative impact of dental caries-one of the most prevalent oral health conditions-on adolescents’ OHRQoL.^27^ Likewise, Bernabé ^4^ reported that approximately 48% of Peruvian children experienced a moderate to very severe impact on their oral health-related quality of life, with the most affected domains being oral hygiene, smiling, and eating. 

The findings of this study reinforce that the presence of these oral conditions, combined with the child’s perception, contributes to the reported impact on quality of life, establishing a logical link with the observed correlation: higher caregiver literacy is associated with lower severity of perceived impact on children.

The strong negative correlation between caregiver literacy and children’s quality-of-life impact supports the hypothesis that better understanding, interpretation, and application of oral health information contribute to reducing disease burden and improving children’s perceived well-being. This result is consistent with findings by Divaris et al. ^5^ who identified parental OHL as a modifying factor in children’s oral health status and quality of life. Although Maito et al.,^25^ did not find a significant relationship between these variables in preschool children, the present study-conducted in older children-suggests that children’s level of autonomy and communication with caregivers may strengthen this association during school age.

In the multivariate analysis, parental OHL was found to have a significant impact on OHRQoL; for each one-point increase in literacy score, the prevalence of its negative impact on quality of life decreased by 12%. This finding is particularly relevant, as efforts to improve caregiver literacy could lead to meaningful reductions in negative outcomes within the field of dental public health. The implementation of educational programs targeting parents and caregivers-especially in vulnerable communities-incorporating content aimed at improving OHL may positively influence children’s oral health status and, consequently, their quality of life during middle childhood.

## LIMITACIONES

Among the limitations of the present study, its cross-sectional design precludes establishing causality between the variables. Additionally, the study population was drawn from a non-governmental organization with relatively specific socioeconomic characteristics, which may limit the generalizability of the findings to other contexts. Nevertheless, the sample size, the high response rate, and the use of validated instruments-such as the HeLD-14 for OHL and the OHRQoL questionnaire-strengthen the internal validity of the study. Future research must consider longitudinal designs, larger sample sizes, and populations with diverse sociodemographic characteristics to allow for more comprehensive analyses of these variables.

## CONCLUSIONES

The findings obtained in this population, considering the limitations of the present study, indicate that higher levels of parental caregiver OHL reduce the severity of the impact on children’s quality of life during middle childhood.

## Source(s) of support:

Self-financed.
